# Clinical Heterogeneity of Guillain-Barré Syndrome in the Emergency Department: Impact on Clinical Outcome

**DOI:** 10.1155/2016/4981274

**Published:** 2016-09-27

**Authors:** Athanasios Papathanasiou, Ioannis Markakis

**Affiliations:** ^1^Department of Neurology, Nottingham University Hospitals NHS Trust, Queen's Medical Centre, Derby Road, Nottingham NG7 2UH, UK; ^2^Department of Neurology, “St. Panteleimon” General State Hospital, 18454 Nikaia, Greece

## Abstract

Guillain-Barré syndrome (GBS) is mainly classified into acute inflammatory demyelinating polyneuropathy (AIDP) and acute motor axonal neuropathy (AMAN). Although diagnosis of GBS requires progressive weakness and universal areflexia or hyporeflexia, cases of GBS with preserved or increased deep tendon reflexes (DTRs) have been increasingly recognized. We report three cases of GBS, presenting at a single unit in six months. Our first case presented with pure sensory symptoms. The second case had nonspecific generalized weakness, while the third presented with typical ascending weakness. One of our patients had preserved DTRs, while the other two had increased DTRs. Our two cases with hyperreflexia were found to have a preceding* Campylobacter jejuni* infection and anti-ganglioside antibodies, and their electrophysiological studies revealed AMAN. The other case had an AIDP. Only one case was offered a diagnosis and treatment from the first emergency department (ED) visit and had a better clinical outcome. Clinical diagnosis of GBS in the ED can be challenging. Delay in diagnosis of GBS in the ED is common due to cases with intact or increased DTRs, atypical pattern of weakness, or pure sensory symptoms. Emergency physicians should be aware of GBS clinical heterogeneity, because early diagnosis and treatment improve clinical outcome.

## 1. Introduction

Weakness and paresthesias are common presenting symptoms in emergency department (ED) [1]. Although Guillain-Barré syndrome (GBS) is an uncommon cause for these chief complaints, early recognition and treatment improve clinical outcome [2]. GBS is an immune-mediated polyneuropathy causing acute neuromuscular paralysis. Clinical diagnostic criteria for GBS, reasserted in 1990, require universal areflexia or hyporeflexia along with progressive weakness [3].

Over the last two decades, the concept of GBS has changed and is currently classified into two major subtypes, acute inflammatory demyelinating polyneuropathy (AIDP) and acute motor axonal neuropathy (AMAN), based on the underlying pathogenesis [4, 5]. There have been studies, describing cases with AMAN that presented with preserved deep tendon reflexes (DTRs), or hyperreflexia [6–9]. It is now well recognized that mainly AMAN and rarely AIDP can present with normal or exaggerated DTRs [10, 11], causing delay in diagnosis and treatment that might affect prognosis [2].

Herein we report three cases of GBS, presenting to our regional neurosciences unit between January and June 2015. We wanted to highlight the clinical heterogeneity of GBS, focusing on cases with preserved or increased DTRs and the impact on clinical outcome.

## 2. Case Reports

### 2.1. Case 1

A 52-year-old female was presented in the emergency department (ED) with one-week history of paresthesias in her hands. Neurological examination was normal and she was discharged by the emergency physicians. Ten days later, she was readmitted with progressive bilateral hand weakness. Examination revealed distal weakness in upper limbs with a Medical Research Council (MRC) scale of 3/5 and preserved DTRs. Sensory examination was normal. Magnetic resonance imaging (MRI) of whole spine was normal, while cerebrospinal fluid (CSF) examination demonstrated increased protein (1.32 g/L) with normal white cell count. Electromyography (EMG) was normal, while nerve conduction studies (NCS) revealed prolonged distal latencies and reduced conduction velocities in upper and lower limbs, suggestive of AIDP. She was commenced on intravenous immunoglobulins (IVIG) for five days. She continued to deteriorate and reached her nadir after two weeks with prominent tetraparesis (MRC grade 2/5). DTRs remained preserved throughout the course of her illness. After six months, she had a modest clinical improvement.

### 2.2. Case 2

A 60-year-old male was presented in the ED for evaluation of ten-day history of mild generalized weakness. Two weeks before presentation, he had diarrhoea that lasted for five days. Examination revealed mild global weakness (MRC grade 4+/5) with hyperreflexia. He was discharged with the impression that he had general malaise due to the preceding gastroenteritis. Ten days later, he was readmitted with progressive weakness in upper and lower limbs. Neurological examination revealed severe distal weakness (MRC grade 2/5) and mild proximal weakness (MRC grade 4/5) in upper and lower limbs. DTRs were brisk symmetrically with downgoing plantar responses. Sensory examination was normal. MRI of brain and whole spine was normal, while CSF examination revealed increased protein (978 mg/L) with normal white cell count. Serum testing showed high titers of anti-GM1 antibody and antibodies to* Campylobacter jejuni*. Sensory NCS were normal, while motor NCS demonstrated markedly reduced distal compound-muscle action potentials in upper and lower limbs. EMG revealed diffuse fibrillation potentials. He was offered the diagnosis of AMAN. He was commenced on IVIG for five days. After six months, his clinical improvement was modest.

### 2.3. Case 3

A 64-year-old male was admitted with one-week history of ascending weakness, following a gastrointestinal infection. Examination revealed severe symmetric tetraparesis (MRC grade 2/5) with hyperreflexia. Plantar responses were downgoing and sensory examination was normal. Brain and whole spine MRI were normal. CSF on admission was normal, while a repetitive CSF after one week demonstrated normal white cell count with increased protein (926 mg/L). Sensory NCS were normal, while motor NCS showed reduced distal compound-muscle action potentials in upper and lower limbs. EMG revealed diffuse fibrillation potentials, rendering the diagnosis of AMAN. Serum testing demonstrated antibodies to* Campylobacter jejuni* and high titers of anti-GM1 antibody. He was commenced on five days of IVIG and after six months he was fully mobilized and independent.

## 3. Discussion

The incidence of GBS is considered to be around 1.72 patients per 100,000 person-years, with an increase of 50% for every 10-year increase in age [12]. Despite the low incidence, GBS is an essential differential diagnosis in the ED of acute onset neurological deficits [2]. Early clinical diagnosis of GBS in the ED can be challenging, due to its clinical heterogeneity. Although clinical diagnostic criteria for GBS require progressive weakness (typically ascending) of more than one limb and universal areflexia or hyporeflexia [3], GBS can present with pure sensory symptoms, descending-asymmetric weakness, and preserved or increased DTRs [1, 2, 10].

One of our patients presented initially with pure sensory symptoms in upper limbs and had a normal neurological examination. She subsequently developed descending weakness, with preserved DTRs throughout the course of her illness. Our second case presented with nonspecific generalized weakness and hyperreflexia following a gastrointestinal infection. Although ascending motor symptoms constitute the typical pattern of GBS (like our third case), presenting symptoms from upper limbs or cranial nerves as well as generalized weakness are often seen [1, 2, 13].

In our first case, DTRs were preserved, while the other two cases had hyperreflexia, making the diagnosis of GBS even more challenging. From a historical point of view, in 1993 was the first report of three North American men who presented with an acute paralytic syndrome following a gastrointestinal infection with preserved or exaggerated DTRs and a pure motor axonal pattern on electrophysiological studies [8]. Around that time, similar cases were described in China [5]. Since then, GBS is mainly subdivided into AMAN and AIDP, according to the underlying immunopathology [4, 5].

Current data suggest that 5% of patients with AIDP and 20% of patients with AMAN have preserved or exaggerated DTRs [11]. Although pure motor involvement might account for the preserved DTRs, it does not explain hyperreflexia [11]. Electrophysiological studies have suggested increased excitability of anterior horn cells due to either upper motor neuron involvement or dysfunction of spinal inhibitory interneurons as the underlying mechanism of hyperreflexia [10, 11, 14].

Over the last two decades, major advances have been made in understanding the underlying immunopathology of AMAN. One of the main underlying mechanisms of AMAN is molecular mimicry of human gangliosides by* Campylobacter jejuni* lipooligosaccharide [11]. AMAN is a primary motor axonal polyneuropathy frequently associated with* Campylobacter jejuni* enteritis and antibodies against gangliosides [10]. Our two AMAN cases had preceding* Campylobacter jejuni* infection and associated anti-GM1 antibodies.

Although GBS is a clinical diagnosis, CSF examination and electrophysiology are integral components of the diagnostic process. Increased CSF protein with normal white cell count (albuminocytologic dissociation) is highly suggestive of GBS. Two of our patients had increased CSF protein with normal white cell count, while one of our cases had normal CSF on admission, which was a timing phenomenon. It is well recognized that one week after symptoms onset the CSF protein will be increased in only 50% of cases, and by the second week the percentage increases up to 80% [15]. Electrophysiology studies in our first case were typical of AIDP, while in the other two cases they were compatible with AMAN [11].

In our small case series, only one of our patients was offered the diagnosis of GBS on the first ED presentation. The other two cases were discharged by the emergency physicians without a Neurology consultation. The diagnosis of GBS was offered after their second admission to the ED by a Neurology resident. After six months, the two cases that have experienced significant delay in diagnosis and treatment were modestly improved, while the other case that had early diagnosis and treatment was fully recovered.

Early diagnosis and prompt treatment of GBS improve clinical outcome and might prevent cardiorespiratory complications that increase morbidity and mortality [1, 2]. Delay in diagnosis or misdiagnosis of GBS in the ED is common. In 2006, McGillicuddy et al. reported that only 5 out of 20 patients with GBS were diagnosed on their first ED presentation [1]. Recently, Dubey et al. demonstrated that, among 69 patients with GBS, the diagnosis was considered during the initial ED presentation in only 34 patients. Moreover, on the first visit, GBS was suspected by the ED physician in only 30,4% of patients [2]. The most common factors associated with failure of considering a diagnosis of GBS were intact or increased DTRs, atypical pattern of weakness, and pure sensory symptoms on presentation [2], which were exactly the same atypical features with our cases. In accordance with our small case series, Dubey et al. identified that higher number of ED visits, delay in diagnosis, and treatment initiation were associated with residual weakness on discharge [2].

In conclusion, early clinical diagnosis of GBS in the ED can be challenging. Cases with preserved or increased DTRs are associated with delay in diagnosis and treatment and have a significant impact on clinical outcome. Herein our small case series highlights the clinical heterogeneity of GBS. Emergency physician's awareness of atypical clinical presentations might improve patient's prognosis.
